# Risk prediction models for discharge disposition in patients with stroke: a systematic review and meta-analysis

**DOI:** 10.3389/fneur.2025.1637606

**Published:** 2025-10-07

**Authors:** Chaoran Xu, Lijun Xiang, Yansi Luo, Li He, Liwen Tai, Yaman Liu, Kaixin He, Min Du, Xiaomei Zhang

**Affiliations:** ^1^Department of Neurology, Nanfang Hospital, Southern Medical University, Guangzhou, China; ^2^School of Nursing, Southern Medical University, Guangzhou, China; ^3^Department of Nursing, Guangzhou Hospital of Integrated Traditional and Western Medicine, Guangzhou, China

**Keywords:** stroke, patient discharge, disposition, risk factors, systematic review, meta-analysis

## Abstract

**Aims:**

Multivariate prediction models can be used to estimate the risk of discharged stroke patients needing a higher level of care. To determine the model’s performance, a systematic evaluation and meta-analysis were performed.

**Methods:**

China National Knowledge Infrastructure (CNKI), Wanfang Database, China Science and Technology Journal Database (VIP), SinoMed, PubMed, Web of Science, CINAHL, and Embase were searched from inception to September 30, 2024. Multiple reviewers independently conducted screening and data extraction. The Prediction Model Risk of Bias Assessment Tool (PROBAST) checklist was used to assess the risk of bias and applicability. All statistical analyses were conducted in Stata 17.0.

**Results:**

A total of 4,059 studies were retrieved, and after the selection process, 14 studies included 22 models were included in this review. The incidence of non-home discharge in stroke patients ranged from 15 to 84.9%. The most frequently used predictors were age, the National Institutes of Health Stroke Scale (NIHSS) score at admission, the Functional Independence Measure (FIM) cognitive function score, and the FIM motor function score. The reported area under the curve (AUC) ranged from 0.75 to 0.95. Quality appraisal was performed. All studies were found to have a high risk of bias, mainly attributable to unsuitable data sources and inadequate reporting of the analytical domain. All statistical analyses were conducted in Stata 17.0. In the meta-analysis, the area under the curve (AUC) value for the five validation models was 0.80 [95%CI (0.75–0.86)].

**Conclusion:**

Research on risk prediction models for stroke patient discharge disposition is still in its initial stages, with a high overall risk of bias and a lack of clinical application, but the model has good predictive performance. Future research should focus on developing highly interpretive, high-performance, easy-to-use machine learning models, enhancing external validation, and driving clinical applications.

**Systematic review registration:**

https://www.crd.york.ac.uk/PROSPERO/, CRD42024576996.

## 1 Introduction

Stroke ranks as the world’s second-deadliest cause and significantly exacerbates long-term impairments and suboptimal rehabilitation (1), placing a heavy societal toll. Due to the high survival rate (2) and the increasing population alongside increased life expectancy, the total count of stroke occurrences is continuously rising (3, 4). About 70–80% of stroke survivors experience varying degrees of functional disabilities, such as language, cognition, swallowing, and limb movement (5). Patients with stroke have relatively high rehabilitation needs and still require further rehabilitation training and care support after discharge (6, 7). Effective rehabilitation nursing is particularly crucial for improving the patients’ quality of life.

Discharge disposition refers to the location or place where a patient will go after being discharged from the hospital, including returning to their original home for home-based care without new family members moving in to provide care, or having a new nanny or caregiver provide care at home, being admitted to the home of relatives for care, or being admitted to rehabilitation institutions, professional care facilities, nursing homes, lower-level hospitals, etc., for non-home-based discharge arrangements (8). If the rehabilitation plan after discharge is not properly implemented and the discharge placement is inappropriate, patients will face multiple challenges such as difficulties in meeting their rehabilitation needs, an increased risk of falls, secondary injuries, and unplanned readmission (9). Therefore, the development of methods for the early identification of patients requiring high levels of care postdischarge has become important for patients, caregivers, clinicians, and payers alike.

Risk prediction assessment tools are used for early screening, identification of delayed discharge or unplanned readmission risk, and early prediction of discharge destination in patients to initiate discharge preparation services as early as possible and shorten the transition time from admission to discharge (10, 11). A systematic review (12) indicates that social economic factors, family support, and the patient’s psychological condition can predict the patient’s discharge placement, and it suggests that clinical healthcare providers should implement personalized discharge plans based on social support, living conditions, insurance type, and the patient’s psychological assessment results. Currently, there is no unified standardized screening tool. The commonly used screening tools are the Blaylock Risk Assessment Screening Score (BRASS) (13) and the LACE Index scoring tool (LACE Index Scoring Tool for Risk Assessment of Death and Readmission) (14), which are widely used to clinically evaluate the risk of delayed discharge or unexpected readmission. However, these measures may not adequately reflect the likelihood of unplanned readmission or delayed discharge in a particular demographic, and they frequently lack specificity for stroke patients. As a result, developing risk prediction models for stroke patients’ discharge disposition has gained importance.

In recent years, many risk prediction models for stroke patients’ discharge disposition have been developed globally, but their usefulness and quality have not been thoroughly assessed. Thus, we embarked on an exhaustive review of pertinent research to meticulously assess the risk bias and clinical feasibility of these models. Our goal was to establish a solid scientific foundation for the creation, implementation, optimization, and tailored prevention and care strategies for risk prediction models concerning discharge outcomes in stroke patients.

## 2 Methods

According to the Preferred Reporting Items for Systematic Reviews and Meta-Analyses (PRISMA) guidelines, this systematic review was conducted. On August 18, 2024, the study protocol was registered on PROSPERO (CRD42024576996).

### 2.1 Search strategy

To cast a wide net in our search, we scoured both Chinese and English databases due to the vast population and widespread use of these languages. The databases under the microscope were the China National Knowledge Infrastructure (CNKI), Wanfang, the China Science and Technology Journal Database (VIP), SinoMed, PubMed, Web of Science, the Cochrane Library, CINAHL, and Embase. Our search was exhaustive, covering the birth of these databases up to September 30, 2024. We combed through with the following search terms: “Stroke,” “Cerebral stroke,” “patient discharge,” “disposition,” “outcome,” “destination,” “prediction model,” “Risk factors,” “Predictors” and “Risk score,” along with their respective variations. The nitty-gritty of our search tactics is detailed in Supplemental material. Moreover, we delved into reference lists and pertinent systematic reviews manually to uncover any pertinent studies that might have been overlooked for inclusion in our analysis.

In conducting our systematic review, we relied on the PICOTS system, which is endorsed by the Critical Appraisal and Data Extraction for Systematic Reviews of Prediction Modeling Studies (CHARMS) checklist (15). This system is instrumental in shaping the review’s objectives, the search methodology, as well as the criteria for including and excluding studies (16). The core components of our systematic review are outlined here:

P (Population): Stroke patients.
I (Intervention model): Published stroke patient discharge disposition risk prediction model construction and/or validation (predictor ≥2).
C (Comparator): No rival model exists.
O (Outcome): Discharge back to home or non-home disposition.
T (Timing): Predictions were derived following an assessment of stroke patients’ fundamental details and laboratory metrics.
S (Setting): Used during hospitalization to predict high-risk discharge disposition for stroke patients, providing personalized interventions to improve patient prognosis and quality of life.

### 2.2 Inclusion and exclusion criteria

The inclusion criteria were: (1) Study subjects: Conforms to the diagnostic standards for stroke guidelines for therapy established by the Neurology Branch of the Chinese Medical Association (17), MRI or CT scans were used to diagnose stroke; aged ≥18 years; (2) Study types: include cohort studies, cross-sectional studies, and case–control studies; (3) Study content: focused on the construction and validation of risk prediction models for discharge disposition in stroke patients; (4) Predict the outcome: discharge to home or non-home setting; (5) Language type: Chinese or English textual form.

The exclusion criteria were: (1) Articles that identified risk factors but did not formulate a predictive model; (2) Study included fewer than two predictors in the model; (3) Studies with incomplete data or full text; (4) reviews, systematic reviews, and meta-analyses; (5) Conference papers.

### 2.3 Study selection and screening

Two writers (XCR and HL) individually imported all retrieved records into Zotero,^1^ an open-source, free reference management software. The duplicate studies were then first removed. Second, the rest of papers were assessed at random based on their abstract and title to see if they qualified for inclusion. The papers were then further examined in full text in accordance with the inclusion and exclusion criteria, and more potentially suitable research were located by looking through the reference lists of all eligible studies. When the authors could not agree on which research were eligible, authors (XCR, HL, and XLJ) talked it out and came to an agreement.

### 2.4 Data extraction

Two authors (XCR and HL) individually extracted search results and checked with each other to minimize bias and ensure the consistency of collected data. According to the CHARMS checklist (Checklists for Critical Appraisal and Data Extraction for Systematic Reviews of Prediction Modeling Studies) (15) used for systematic review data extraction of risk prediction models, the following data were extracted from the included studies: basic information included details such as author, publication year, research design, participants, data source, and sample size. Variable selection method, model building methodologies, validation kinds, performance measures, continuous variable handling techniques, final predicted variables, and presentation styles are all examples of model information. A third reviewer (XLJ) confirmed the information gathered during data extraction. To make sure they all agreed, the three researchers discussed and worked out any discrepancies. The two reviewers had extremely high consistency during the whole-text screening phase, with a Cohen’s kappa coefficient of 0.86 [Cohen’s kappa≥0.75 indicates extremely good consistency in the screening results (18)].

### 2.5 Risk of bias and applicability assessment

The quality of the included studies was assessed based on the TRIPOD (Transparent Reporting of a multivariable prediction model for Individual Prognosis Or Diagnosis) guidelines (19), which offer a template for the transparent and comprehensive reporting of a multivariate predictive model for individual prognosis or diagnosis. Furthermore, the PROBAST (Predictive Model Risk of Bias Assessment Tool) was used to assess the risk of bias and applicability of the included studies in the present research on predictive modeling (20). The risk of bias was assessed in four domains: participants, predictors, outcomes, and analysis methods. Each domain comprised 2–9 questions, and the answers were scored as “Yes/Likely,” “No/Unlikely,” or “No Information,” representing low risk of bias, high risk of bias, or unclear risk of bias, respectively. The domain of risk of bias was judged as low risk if all questions were scored as low risk; if one or more questions were scored as unclear, the domain was judged as unclear; and if one or more questions were scored as high risk, the domain was judged as high risk. Applicability was assessed in three fields—“participants,” “predictors” and “outcome measures” (excluding the critical issues)—with criteria similar to those used for the bias assessment (21, 22). Two researchers (XCR and HL) used the PROBAST tool to independently assess the risk of bias and the applicability of the prediction models included in the present study. A third researcher (XLJ) was consulted for a conclusion in cases of disagreement.

### 2.6 Data synthesis and statistical analysis

Use Stata 17.0 software to perform analysis using the area under the curve (AUC) and 95% confidence interval (CI) as effect statistics. Employ the Q test and *I^2^* statistic to assess heterogeneity among multiple studies. If *p* > 0.1 or *I^2^* < 50%, it indicates low heterogeneity between studies, and a fixed-effects model is used for combined analysis (23); if *p* ≤ 0.1 or *I^2^* ≥ 50%, it suggests significant heterogeneity among studies, and a random-effects model is adopted, along with sensitivity analysis (24). To assess publication bias, Egger’s test and a funnel plot were employed. At *α* = 0.05, the significance level is established. There is less chance of publication bias when *p* > 0.05.

### 2.7 Ethical approval

Ethical approval was unnecessary in this study, because it was a systematic review of existing articles, and no individual patient data were handled.

## 3 Results

### 3.1 Study selection

**Figure 1:**
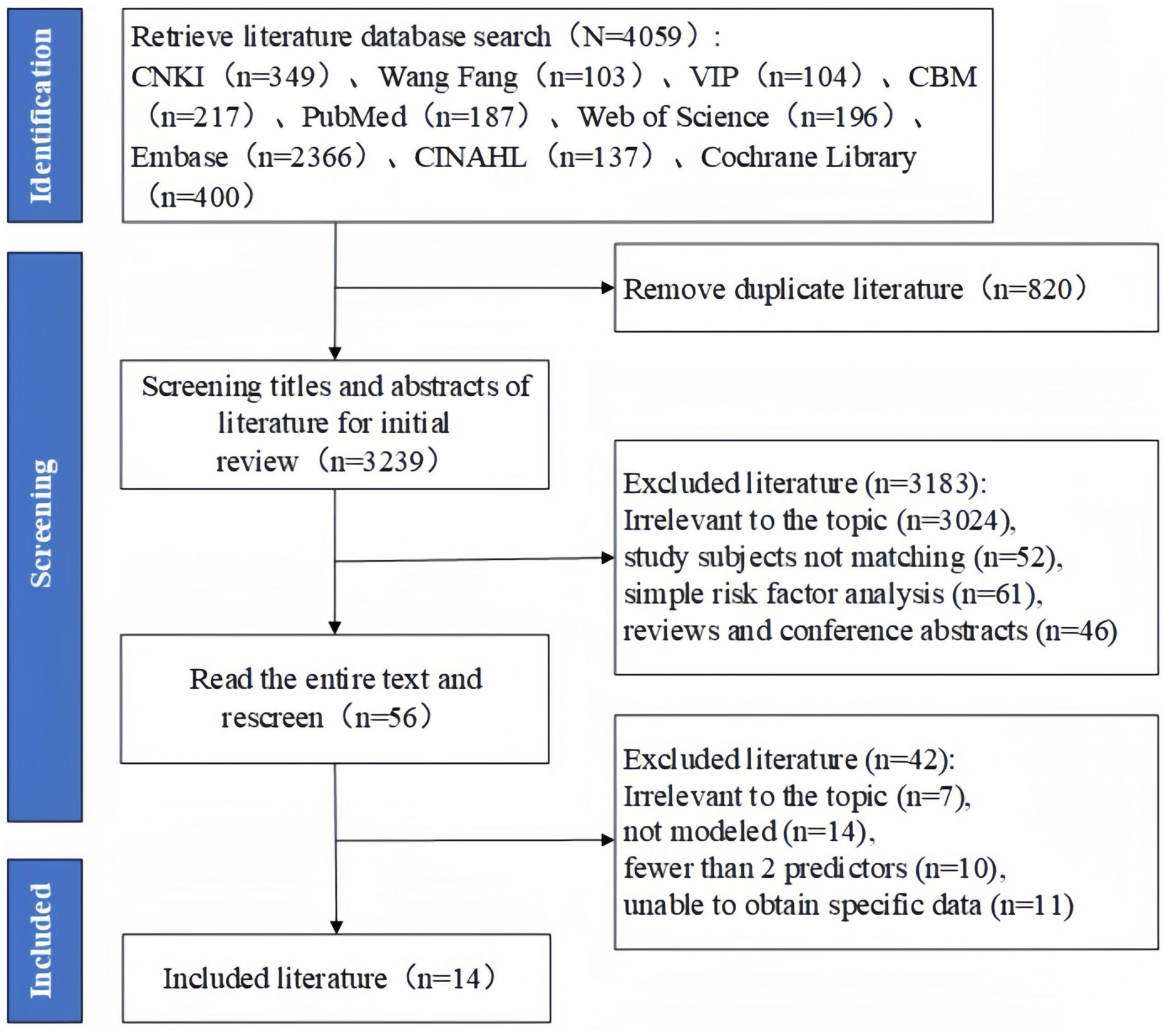
Preferred Reporting Items for Systematic reviews and Meta-Analyses (PRISMA) flowchart of literature search and selection.

A total of 4,059 indexed documents were found in the first search. The titles and abstracts of 3,239 documents were evaluated after 820 duplicate records discovered in all databases were eliminated. Fifty six papers were included for additional review after this screening procedure. In the course of the following assessment, 15 studies were ruled out because they either did not create prediction models or only addressed risk factors. Furthermore, seven studies were deemed incompatible with the review’s target population, 10 studies included fewer than two predictors, and 11 studies were unable to provide necessary data. The PRISMA flow diagram, which depicts the thorough literature screening procedure and outcomes, is displayed in Figure 1.

### 3.2 Study characteristics

The review’s studies were released between 2008 and 2024. Four of these were carried out in Japan, three in the United States, two in Australia, and one each in China, the United Kingdom, Switzerland, South Korea, and France. Regarding study design, six were prospective studies (including two multicenter studies), and eight were retrospective studies. The subjects consisted of two categories, with nine studies focused on stroke patients in the acute phase and five studies focusing on stroke patients undergoing rehabilitation in the sub-acute phase. Predicting discharge outcomes mainly involves two scenarios: home and non-home discharge. The former includes patients being discharged back to their own homes or the homes of relatives or friends, while the latter includes patients dying during hospitalization or being discharged to inpatient rehabilitation facilities, professional nursing facilities, nursing homes, secondary hospitals, rehabilitation centers, etc. The incidence of home discharge ranges from 15.1 to 85%, and the incidence of non-home discharge ranges from 15 to 84.9%. The number of people included in these studies varied, with sample sizes ranging from 296 to 74,425. Table 1 provides specifics.

**Table 1:** Overview of basic data of the included studies.

Included literature	Year of publication	Country	Study design	Object of study	Research source	Data sources	Sample size (s)			Forecast the outcome	Outcome event ratio (%) (Home/non-home)
							Totality	Development	Validate		
Lensky et al.	2024	Australia	Retrospective cohort study	Acute stroke patients	The Canberra Hospital	Clinical records	296	–	–	Home; non-home (rehabilitation institutions, nursing homes, death)	67.5/32.5
Cui et al.	2024	China	Prospective observational study	stroke patients	Neurology Department at a university hospital	the hospital electronic medical record system and through face-to-face consultations with the patients or their caregivers	523	366	157	Home; non-home (inpatient rehabilitation institutions, professional nursing institutions, nursing homes, subordinate hospitals, rehabilitation centers)	69.98/30.01
Veerbeek et al.	2022	Switzerland	prospective cohort study	Acute stroke patients	the Stroke Center of the Neurology Department of the Luzerner Kantonsspital, Lucerne, Switzerland	a local registry	953	121	832	Home; non-home (rehabilitation center, nursing home, other hospitals, care facilities, death)	36/64
Ito et al.	2022	Japan	Retrospective cohort study	Stroke patients	Tokyo Bay Rehabilitation Hospital	Medical chart notes	1,229	–	–	Home; rehabilitation facility	82.3/17.7
Itaya et al.	2022	Japan	Prospective cohort study	Acute stroke patients	A stroke center in Japan	The electronic health record (EHR) database in the medical center	1,214	254	960	Home; non-home (rehabilitation facility, nursing home, other hospital, death)	51/49
Cho et al.	2021	United States	Retrospective cohort study	Acute stroke patients	The Centers for Medicare and Medicaid Services (CMS)	Electronic Dataset	74,425	–	66,172	Home; non-home (rehabilitation facility, other hospital, death)	42.5/57.5
Berker et al.	2020	England	Prospective cohort study	Acute stroke patients	The acute stroke unit at the University Hospital of Wales	An existing, anonymized database	1,131	1,016	115	Home; non-home (death, community hospital, other hospital)	15.1/84.9
Kubo et al.	2020	Japan	Retrospective cohort study	Acute stroke patients	37 acute hospitals	The Japan Rehabilitation Database (JRD)	4,216	2,810	1,406	Home; non-home (rehabilitation facilities, other hospitals, care facilities)	52/48
Kim et al.	2020	South Korea	Multicenter prospective cohort study	Moderate stroke patients	The Korean Stroke Cohort for Functioning and Rehabilitation (KOSCO)	Medical chart notes	732	–	–	Home; rehabilitation facility	51/49
Itaya et al.	2017	Japan	Retrospective cohort study	Acute stroke patients	A stroke center in Japan	The electronic medical record database	3,200	2,240	960	Home; non-home (death, rehabilitation facilities, other hospitals, care facilities)	48/52
Ouellette et al.	2015	United States	Retrospective cohort study	Acute stroke patients	An inpatient rehabilitation facility	Electronic data source	407	–	–	Home; rehabilitation facility	73/27
Béjot et al.	2015	France	Retrospective cohort study	Ischemic stroke patients	The population-based Dijon Stroke Registry	Electronic data source	980	–	–	Home; non-home (an inpatient rehabilitation institution, a convalescent home, a long-term nursing facility)	53.3/46.7
Stineman et al.	2014	United States	Retrospective cohort study	Acute stroke patients	110 Veterans Affairs (VA) Medical Center	The Electronic medical record database	6,515	3,909	2,606	Home; non-home (death, rehabilitation facilities, other hospitals, care facilities)	85/15
Brauer et al.	2008	Australia	Multicenter prospective cohort study	Stroke patients	15 rehabilitation units	Medical chart notes	554	–	–	Home; non-home (hostel, nursing home)	74/26

### 3.3 Basic characteristics of prediction models

A total of 22 prediction models were constructed across the 14 studies. Cui et al. (25) developed 6 models and before deciding on the best one for creating nomograms, Lensky et al. (26) developed 3 models, Brauer et al. (7) developed 2 models; 1 model was built in each of the other studies. In terms of variable selection, Cui et al. (25) (8.3%) optimal model used the Feature Importance analysis, Lensky et al. (26) (8.3%) applied principal component analysis, Itaya et al. (27) and Stineman et al. (28) (16.7%) applied the backward variable selection method, Brauer et al. (7) (8.3%) used a multinomial forward stepwise logistic regression, and the other 7 studies (58.3%) utilized univariate analysis to select variables. Regarding modeling methods, Cui et al. (25), Lensky et al. (26), Veerbeek et al. (29), and Berker et al. (30) employed machine learning method, such as the support vector machine (SVM), random forests (RF), naive bayes (NB), gradient boosting (GB), KNearest Neighbors (KNN) (25), AdaBoost, Bootstrap (26), and CART decision tree (29), while other 10 models used logistic regression (LR). The performance indicators mentioned in this research report include model Calibration, sensitivity, specificity, accuracy rate and AUC. For all these indicators, the closer the value is to 1.0, the better the performance. Thirteen models provided the area under the curve (AUC), which varied between 0.75 and 0.95; one model (31) reported the C statistic. Nine studies addressed calibration, and the most commonly used approach was the Hosmer-Lemeshow test. Only one study (25) compared machine learning algorithms with other classical statistical methods (including logistic regression). Cui et al. (25) compared the LR, SVM, RF, NB, GB, and KNN models using data from 523 stroke patients and found that the RF model was the best. Additionally, the AUC values of four of the machine learning algorithms were higher than that of the logistic regression model, while the KNN model was slightly lower than the LR model (LR: 0.85, SVM: 0.92; RF: 0.95; NB: 0.93; GB: 0.93; KNN: 0.84). Details are provided in Tables 2, 3.

**Table 2:** Overview of the information of the included prediction models.

Included literature	Models validation		Modeling Methods	Modeling/Validation Quantity (s)	Variable the choice of	Treatment method of continuous variables	Model performance (modeling/validation)					Number of missing values (s)	Missing value processing method
	interior	outside					Discrimination[95% confidence interval]	Calibration	sensitivity	specificity	accuracy rate		
Lensky et al. (30)	Cross validation	–	Machine learning method: KNN、AdaBoost、Bootstrap	3	Principal component analysis	Partially converted to categorical variables	–	–	–	–	KNN:0.817/−AdaBoost:0.90/−Bootstrap:0.804/−	2,424	Remove directly
Cui et al. (25)	Random split	–	Machine learning method: LR, SVM, RF, NB, GB, KNN	6	Optimal model: Feature Importance analysis	Partially converted to categorical variables	AUC: LR:0.85/− SVM:0.92/− RF:0.95/− NB:0.93/− GB:0.93/− KNN:0.84/−	H–L: RF:−/0.049	LR:0.894/− SVM:0.830/− RF:0.809/− NB:0.851/− GB:0.872/− KNN:0.745/−	LR:0.845/− SVM:0.855/− RF:0.900/− NB:0.845/− GB:0.882/− KNN:0.891/−	LR:0.85/− SVM:0.84/− RF:0.87/− NB:0.84/− GB:0.87/− KNN:0.84/−	3	Remove directly
Veerbeek et al. (29)	A 10-fold cross-over validation	External validation	Machine learning method: CART decision tree	1	Univariate analysis	Keep continuity	AUC:0.84[0.76 ~ 0.91]/0.74[0.72 ~ 0.77]	–	0.70/0.59	0.97/0.90	0.88/0.77	167	Multiple attribution method
Ito et al. (33)	–	–	Logistic regression	1	Univariate analysis	Keep continuity	–	H–L:0.944/−	–	–	0.883/−	44	Remove directly
Itaya et al. (36)	–	Time verification	Logistic regression	1	–	–	AUC:−/0.80[0.77 ~ 0.82]	calibration curve	−/0.91	−/0.59	–	–	–
Cho et al. (11)	Nonrandom split	–	Logistic regression	1	Univariate analysis	Partially converted to categorical variables	–	–	–	–	–	1,310,939	Remove directly
de Berker et al. (27)	100-fold cross-validation	–	Machine learning method: RF	1	Univariate analysis	–	–	–	0.78/−	–	0.704/−	11	Remove directly
Kubo et al. (37)	Random split	–	Logistic regression	1	Univariate analysis	Keep continuity	AUC:0.88/0.86	H–L:0.510/−	0.804/0.782	0.803/0.785	–	–	–
Kim et al. (32)	–	External validation	Logistic regression	1	Univariate analysis	Partially converted to categorical variables	AUC: 0.87[0.84 ~ 0.90]/0.86[0.80 ~ 0.92]	H–L:0.405/−	0.87/0.835	0.862/0.833	–	833	Remove directly
Itaya et al. (27)	Random split	-	Logistic regression	1	Backward variable selection method	Keep continuity	AUC:0.88[0.86 ~ 0.89]/0.87[0.85 ~ 0.89]	H–L:0.26/−	85.0/88.0	75.3/68.7	–	0	Complete case analysis
Ouellette et al. (34)	–	–	Logistic regression	1	Univariate analysis	–	AUC:0.76/−	–	0.76/−	0.64/−	–	–	–
Béjot et al. (31)	–	External validation	Logistic regression	1	–	–	C statistic:−/0.75[0.72–0.78]	H–L: −/0.35	−/0.570	−/0.812	–	95	Remove directly
Stineman et al. (28)	Random split	–	Logistic regression	1	Backward variable selection method	Partially converted to categorical variables	AUC: 0.82/ 0.80	H–L: 0.23/0.30	–	–	–	–	–
Brauer et al. (7)	10-fold cross-validation	–	Logistic regression	2	Multinomial forward stepwise logistic regression	Partially converted to categorical variables	–	Model 1: Nagelkerke r^2^ = 0.405/− Adjusted Model 2: Nagelkerke r^2^ = 0.386/−	Model 1:0.99/− Adjusted Model 2: 0.995/−	Model 1:0.333/− Adjusted Model 2: 0.195/−	Model 1:0.873/0.864 Adjusted Model 2:0.852/ 0.835	12	Remove directly

“–”: not reported. LR, Logistic regression; SVM, support vector machine algorithm; RF, random forest; NB, naive bayes; GB, gradient boosting; KNN, K-nearest neighbor algorithm; AdaBoost, Adaptive improvement; Bootstrap, bagging bag-algorithm; Nagelkerke r^2^, Pseudo-R^2^, Used to assess the fit of the model overall, The closer the value is to 1, the better the model fit; Considering Nagelkerke r^2^ = 0–0.2 as the differential resolution, 0.2–0.4 for moderate resolution, 0.4–0.6 for a better resolution, 0.6–0.8 for very good, The term “0.8–1” is excellent. The AUC (Area Under the Curve) is the area under the subject’s operating characteristic curve. We considered AUC = 0.5–0.7 as poor resolution, 0.7–0.8 as moderate resolution, 0.8–0.9 as good resolution, and 0.9–1.0 as excellent. H-L: Hosmer-Lemeshow good of fittest.

**Table 3:** The frequency of each variable selection method.

Treatment method of continuous variables	N(%)
Principal component analysis	1(8.3)
Optimal model: Feature Importance analysis	1(8.3)
Univariate analysis	7(58.3)
Backward variable selection method	2(16.7)
Multinomial forward stepwise logistic regression	1(8.3)

The prediction models in the majority of the 14 investigations were validated either internally or externally, proving their generalizability and robustness. In particular, nine research were validated internally, while four studies were validated externally. Two articles were combined with internal and external validation, and two articles did not perform any validation after model construction. Regarding model presentation, 7 used risk scoring system, 5 used formula of risk score obtained by regression coefficient of each factor, and 1 studies used nomograms. The total amount of predictors that are included ranges from two to ten. The most often utilized factors in these models are age, the Functional Independence Measure (FIM) cognitive function score, the National Institutes of Health Stroke Scale (NIHSS) score at admission, and the FIM motor function score, which appear in six, five, five, and four studies, respectively. Other commonly used predictive indicators include the modified Rankin Scale (mRS) score at admission and stroke types, which appear in three studies. Barthel index (BI), Enteral feeding, pre-admission residence, marital status, paralysis degree, living alone and comorbidities, which appear in two studies. Additional details are available in Table 4.

**Table 4:** Includes the literature predictors and the presentation methods.

Included literature	Number of predictors (s)	Model predictors	Model presentation
Lensky et al. (30)	5	The mRS score, SSS score, NIHSS score, dyslipidemia, and hypertension	Formula of risk score obtained by regression coefficient of each factor
Cui et al. (25)	10	NIHSS Score, household income, BI score, FS score, risk of falls, risk of stress injury, tube feeding, depression, age, and WST score	Nomogram model
Veerbeek et al. (29)	3	ADL score, motor function (Short-LIMOS-1), Cognitive and Communication function (Short-LIMOS-2)	Formula of risk score obtained by regression coefficient of each factor
Ito et al. (33)	6	Age, duration from stroke onset to admission, solitary or not, MMSE score, FIM motor function score, and FIM cognitive function score	Formula of risk score obtained by regression coefficient of each factor
Cho et al. (11)	5	Source of admission (referral from professional nursing institution, other), comorbidities (acute heart attack, cerebral hemorrhage), age	The regression coefficients were converted to a risk-scoring system
de Berker et al. (27)	2	NIHSS score and mRS score	Formula of risk score obtained by regression coefficient of each factor
Kubo et al. (37)	6	Age, type of stroke, degree of paralysis, mRS score, NIHSS score, and BI score	Risk scoring system
Kim et al. (32)	4	FIM scores of cognitive function, FAC score, CCI score, and marital status	Selection factors were analyzed by logistic regression to generate a weighted scoring system
Itaya et al. (27)	5	Living alone, stroke type, FIM motor function score, FIM cognitive function score, degree of paralysis	Risk scoring system
Ouellette et al. (34)	8	STREAM Score, FIM motor function score, total FIM, FIM bed transfer, FIM toilet, FIM bathing, FIM bladder management, FIM memory	Risk scoring system
Béjot et al. (31)	10	Age, NIHSS score, gender, pre-admission level of self-care, comorbidities (cancer, renal dialysis), risk factors (atrial fibrillation, congestive heart failure), stroke type, admission blood glucose level.	Risk scoring system
Stineman et al. (28)	8	Marital status, pre-admission residence, FIM motor function score, FIM cognitive function score, comorbidities (liver disease), mechanical ventilation, tube feeding, ICU admission	Risk scoring system
Brauer et al. (7)	4	From supine to lateral lying ability (MAS-1), walking ability (MAS-5), age, residence before admission	Formula of risk score obtained by regression coefficient of each factor

mRS, Modified Rankin Scale, Modified Rankin scale; SSS, Scandinavian Stroke Scale, The Scandinavian Stroke Scale; NIHSS, National Institutes of Health Institute Stroke Scale, The US National Institute of Health Stroke Score Scale; MMSE, Mini-Mental State Examination, Simple mental state scale; BI, Barthel index, The Pap index; FS, the FRAIL Scale, The Frailty Screening Scale; WST, Water-Swallow Test, Wa field drinking water test; FIM, Functional Independence Measure, Functional independence evaluation and measurement table; ADL, Activity of Daily Living, Ability of daily living activities; CCI, Charlson comorbidity index, Charlson Comorbidity Index; STREAM, Stroke Rehabilitation Assessment of Movement, Motor function scale of stroke rehabilitation; MAS, Motor Assessment scale, Motor function assessment scale of stroke patients; FAC, Functional ambulation category scale, functional ambulation category; Short-LIMOS, A short version of the multidisciplinary-based observation scale for the Lucerne ICF (48).

### 3.4 Results of quality assessment

#### 3.4.1 Risk of bias assessment

**Figure 2:**
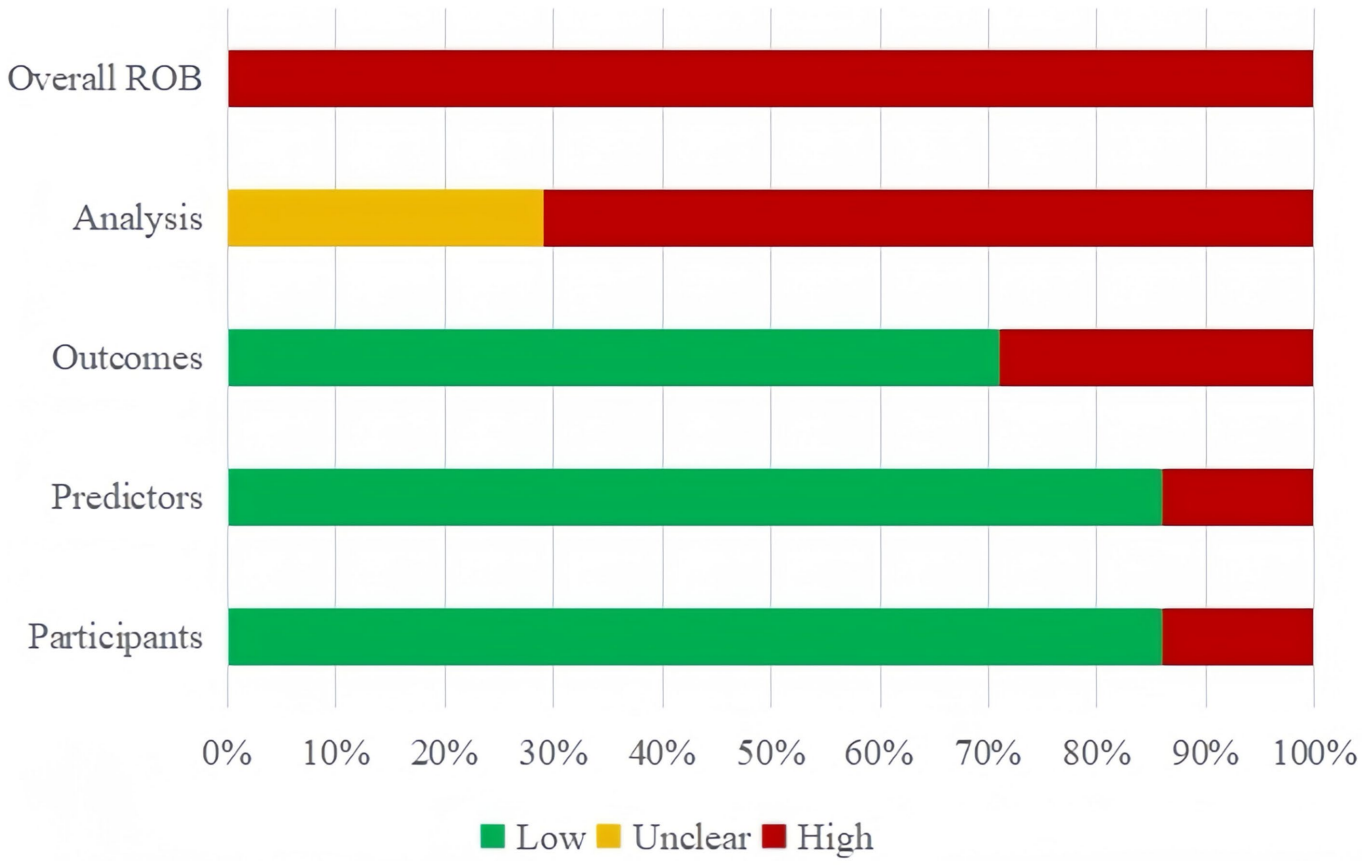
Risk of bias assessment for the predictive model studies.

All 14 studies included in the narrative review were scored as risk of bias (ROB) using the PROBAST (Figure 2). All 14 (100%) of the studies were assessed as having a high ROB overall, pointing to methodological issues with the development or validation procedures. The main reasons for high ROB were as follows: events per variable (EPV) < 10, defects or omission in handling missing data, flaws in methods for the selection of predictors and inadequate assessment of model performance measures.

In the participant domain, two studies were found to have a significant risk of bias, mainly as a result of unreliable data sources (10, 32). In the predictor domain, two studies were judged to have a high risk of bias because they included predictive variables that were based on hypotheses (29, 33). In the outcome domain, four studies were identified as having a high risk of bias due to an inappropriate time interval being left between evaluating predictive factors and determining outcomes (7, 11, 32, 33).

All 14 studies were judged to have a high risk of bias in the analytical domain. Five of these studies lacked sufficient sample size for validation or modeling (7, 11, 29, 32, 34). For modeling studies, the number of EPV should be greater than 20 for each variable to avoid over-fitting of the model; for model validation studies, at least 100 subjects with outcome events should be included (35). Four studies lacked detailed information on participant follow-up, withdrawal, or study termination, and how missing data were handled (28, 34, 36, 37),six studies directly deleted subjects with missing values, without adopting the complete case analysis method to reduce bias (7, 11, 25, 31–33). Continuous variables were transformed into categorical variables in six studies (7, 11, 25, 26, 28, 32), which led to a substantial loss of information and even diminished the model’s capacity for prediction. Five research failed to thoroughly evaluate their prediction models’ predictive capabilities (7, 11, 26, 30, 33). Four studies used the randomized split-validation method (25, 28, 36, 37), and one did not specify the exact validation method (11). Details are provided in Supplementary Table S2.

#### 3.4.2 Applicability assessment

Twelve studies demonstrated generally good applicability, while the remaining two studies (28, 32) showed poor applicability: In the Participants domain, Kim’s study focused on home discharge after subacute rehabilitation of moderate stroke patients, while Stineman’s study restricted participants to veteran subjects, which limited the studies’ generalizability. Overall, the results of this systematic review indicated a high risk of bias across all studies, which raises the possibility of methodological issues with the models’ creation or validation. Details are provided in Supplementary Table S2.

### 3.5 Quality assessment of the literature

The overall quality of the studies included in this analysis was rather excellent, covering more than 70% of the reporting items, according to our quality evaluation, which was based on the TRIPOD guidelines (19). Nevertheless, we also observed certain restrictions or ambiguities in specific reporting elements. For example, despite Lensky’s study (26) being comprehensive, it is deficient in providing sufficient details about its predictive model parameters and their applications. In their comprehensive report, Cho’s study (11) failed to define the parameters of their prediction model or the methodology for determining the sample size. Despite the fact that the TRIPOD rules offer us a reliable instrument for evaluating the quality of predictive models, this evaluation might not adequately capture all the information essential to predictive modeling because of the models’ complexity and diversity. For instance, sample size has a big impact on how accurate and robust models are, and choosing and modifying model parameters is essential for predictive power and model optimization. Detailed quality assessment in Supplementary Table S6.

### 3.6 Meta-analysis of validation models

**Figure 3:**
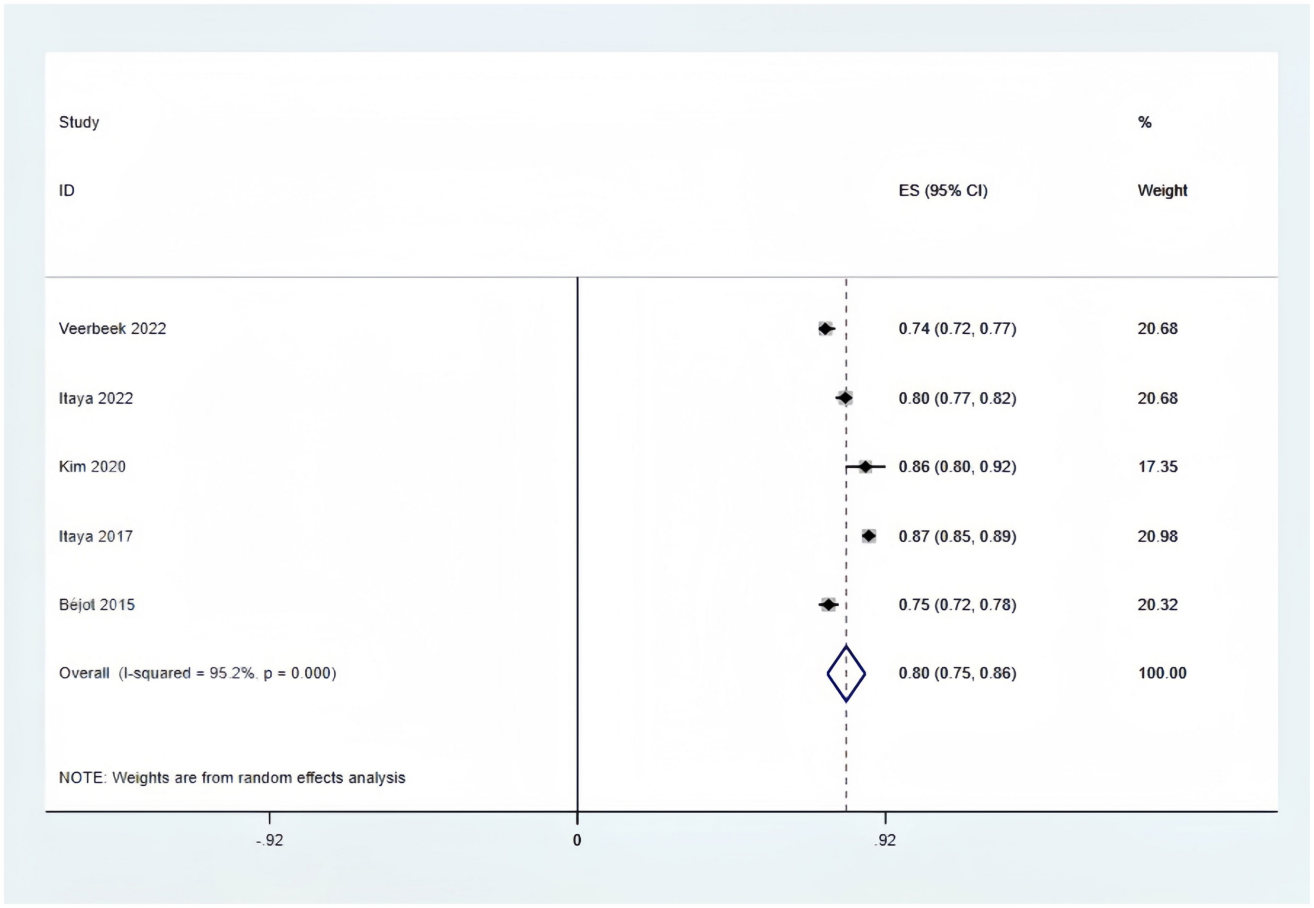
Forest plot of pooled AUC estimates for validation models.

**Figure 4:**
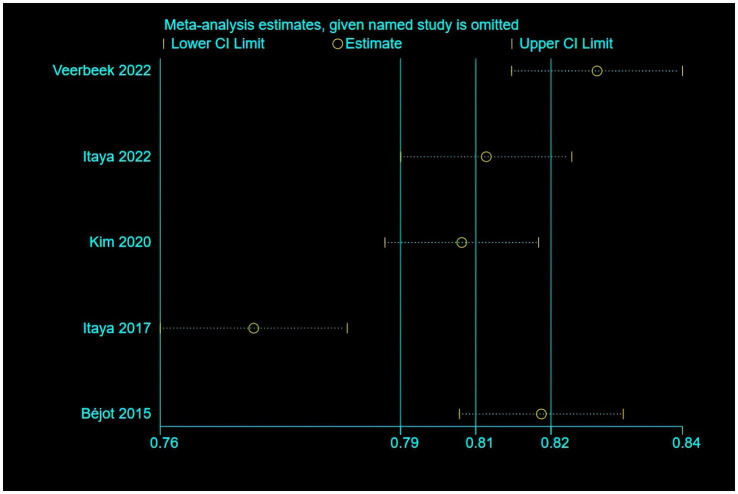
The results of the sensitivity analysis of the model.

**Figure 5:**
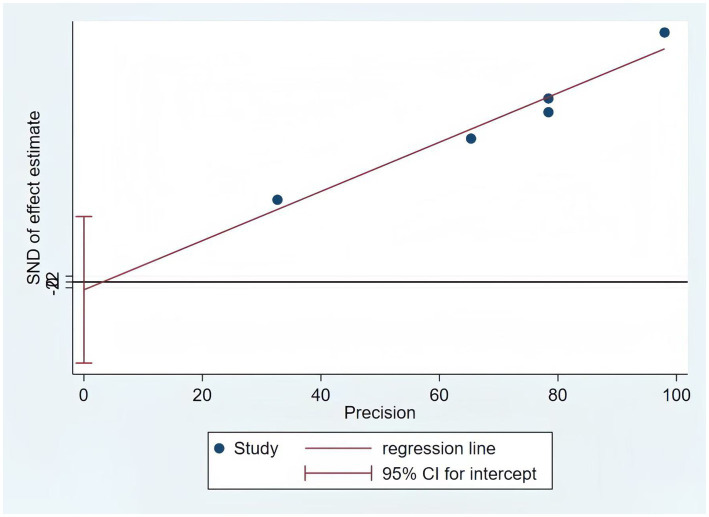
The results of the Egger test of the model.

Only five of the listed papers were suitable for synthesis because of inadequate reporting on the model development specifics. The validation model used a random effects model to calculate the combined AUC, which was 0.80 (95% CI [0.75–0.86]), indicating good overall predictive performance (Figure 3). The heterogeneity test revealed significant heterogeneity (*p* < 0.001, I^2^ = 95.2%), and a random effects model was used. Changes in study design, sample size, predictor selection, and outcome definition may be the cause of the significant variability among studies. The findings’ robustness was demonstrated by the results’ stability even after any one study was eliminated. In addition, the sensitivity analysis confirmed the robustness of the results, with no single study altering the magnitude of the combined effect (Figure 4). The Egger’s test indicated no significant asymmetry (*p* = 0.757) (Figure 5). The funnel plot showed a roughly symmetrical distribution of the scatter points around the pooled effect size, with all points falling within the pseudo 95% confidence limits (Supplementary Figure 1), suggesting a low likelihood of publication bias. The meta-regression analysis identified the country of the participants (*p* = 0.5235), to be not a significant source of the heterogeneity.

## 4 Discussion

Stroke disease is characterized by a “high disability rate,” often leaving stroke patients with disabilities of various aspects and degrees after receiving acute treatment (6) Assisting stroke patients in safely transitioning from the hospital to home has become a focal point of clinical nursing work. At the same time, with the increase in hospital bed turnover rates, the average length of stay for stroke patients has significantly shortened. Factors such as the patient’s advanced age, mobility difficulties, or memory decline often result in discharge before full recovery, shifting the significant responsibility of continued rehabilitation post-discharge to community medical institutions, patients, and their families (38, 39). Therefore, accurately assessing the non-home discharge risks for stroke patients, as well as early detection and precise intervention, is essential to lower the frequency and severity of negative outcomes.

### 4.1 Models perform well, but there is a high risk of bias; external validation and various modeling are essential

Following a thorough screening and search of the literature, we found 14 original research. The risk prediction algorithms now in use for stroke patients’ discharge disposition are still in the early stages of development. All of these investigations included model construction and showed strong performance, with AUC values above the 0.7 threshold and ranging from 0.75 to 0.95. It is noteworthy that 2 studies (27, 29, 36) performed internal and external validation. This step is essential for assessing the generalization ability of the model, detecting overfitting, boosting prediction accuracy, and guaranteeing the dependability and significance of the findings (40). AUC values of 0.80 (95% CI [0.75–0.86]) were also noted for validation models, coupled with notable heterogeneity that was probably brought on by different demographic features, predictive factors, and methodology. Furthermore, most of the included studies presented their model results in the form of a risk-scoring system. The simplified scoring system is relatively simple and easy to understand clinically, and can achieve intuitive, convenient and effective individualized risk prediction, thus promoting the clinical application of healthcare professionals (41). Overall, these models have strong predictive power for discharge non-home disposition of stroke patients, showing favorable performance. However, the model’s clinical application was limited because all analyzed papers were deemed to have a high bias risk based on the PROBAST checklist.

First, the data sources are biased. Eight of the studies were retrospective analyses, which perhaps increased recollection bias and impacted the model’s quality. The majority of the data came from single-center research with small sample sizes. Multicenter, large sample, high-quality prospective studies should be given priority in future research in order to reduce recollection bias and enable the adaptation of prediction models to a larger patient population.

Second, there is also a bias in the model construction. (1) In terms of variable selection, univariate selection was one of the most commonly employed methods (58.3% of studies), and it ignored the interaction between variables and potential problems of collinearity, which may lead to inaccurate prediction results (42). (2) Eight research have explicitly eliminated missing data, and four studies have not reported missing values. The link between predictor factors and outcomes may become biased as a result of this approach; even in the absence of bias, missing data reduces precision and increases the confidence interval (43). (3) Ten research built their models using logistic regression, which limited their capacity to capture intricate interactions between variables and impacted the model’s accuracy and stability. We propose that more sophisticated variable screening techniques, including LASSO regression, which can address the issue of multicollinearity among variables and assist in identifying the most predictive variables (44), be used to increase the model’s stability and predictive power. In order to lessen the negative effects of missing data on statistical analysis and model stability, techniques like multiple imputation and single imputation should be applied when addressing missing data. Cui et al. (25), Lensky et al. (26), Veerbeek et al. (29), and Berker et al. (30) developed models using machine learning methods in this systematic review. Despite their potential to enhance prediction accuracy (45), machine learning algorithms showed limited advantages in this review. We assume that variables like random data set division, univariate analysis-based variable screening methods, and inadequate sample size may be connected to this phenomenon.

Finally, there are some limitations to the model validation. The predictive power of the model may be influenced by population and regional differences, thus highlighting the need for comprehensive validation during model development. For example, Kim et al. (32) study used a high sample size and a prospective cohort design, but it omitted internal validation. While random split validation is typically thought of as an internal validation method, it does not address the matter of model overfitting (46). Furthermore, only four studies performed external validation, and the remaining articles lacked such validation, limiting the generalization and applicability of the model (47). To increase the model’s generalizability, future research should place a strong emphasis on external validation, particularly across various geographies, racial groups, cultural contexts, and lifestyle characteristics. Different phases of the disease and variations in stroke types (such as hemorrhagic, mixed, and ischemic strokes) should also be taken into account. The degree of social support, frailty, and treatment strategy may also affect predictive performance. Taking all of these things into account will help to increase the model’s applicability and dependability.

### 4.2 Dissimilarities and similarities among predictors: emphasize age, NIHSS, FIM cognitive and motor function

Between two and ten predictors were included in the 22 models in this study, which were primarily divided into four groups: Sociodemographic factors: (e.g., age, marital status, residence before admission, lived alone), Clinical factors: (e.g., type of stroke, degree of paralysis, mRS, NIHSS, motor cognitive function score), Psychological factors: (e.g., depression), Comorbidities: (e.g., dyslipidemia, high blood pressure, acute heart attack, cerebral hemorrhage), Treatment: (e.g., mechanical ventilation, tube feeding, ICU admission). Some similarities were found despite differences in predictor selection brought about by research types and included variables. Age, NIHSS as well as FIM cognitive function and motor function scores at admission, are high-frequency predictive factors. Age was important predictor of institutional long-term care admission directly from the hospital after an acute stroke. Multiple studies (11, 25, 31, 33) have found a positive association between age and discharge non-home placement, where older patients may have more comorbidities with less support at home and may require further medical care and monitoring at institutions. Studies reported a strong correlation between non-home firing and initial neurological function as assessed by the NIHSS score (25, 28, 30, 31). High NIHSS scores are a common risk factor for neurological deficit after severe stroke and poor recovery after stroke. FIM motor and cognitive scores at admission showed significantly high score weights, with the former showing the highest predictive power in the model (30), a finding supported by Stineman et al. (28) Instead, Kim et al. (32) and Choi-Kwon et al. (11) state that cognitive FIM is the most important factor. This may be because patients with low cognitive ability often struggle to self-discharge or reunite with family, as cognitive and emotional disorders impose a greater burden on caregivers than physical disability. Further study is required to evaluate the effectiveness of the place where a patient gets referred at discharge, and discharge decisions should take into account patient-specific biopsychosocial characteristics that may take precedence over isolated outcomes of the outcome measures.

Therefore, in order to promptly identify high-risk patients, early screening should concentrate on these common variables. Nursing intervention is limited, though, as this study also revealed that many of the present factors are hard to directly alter. Future studies should look into incorporating controllable characteristics such stroke knowledge, psychological state and medication adherence in an effort to achieve individualized care interventions and prevent needless hospitalizations and resource misallocation.

### 4.3 Implications for clinical practice

Applying credible models helps to identify patients who require non-home discharge to higher levels of care, enabling early intervention plans. To guarantee the precision and legitimacy of the best outcomes, future research efforts should follow the TRIPOD guidelines. This means that investigators should prioritize the implementation of studies with a larger sample size and adopt a prospective study design. Numerous included research had difficulties with sample size, selecting predictors, and handling continuous variables. The application of sophisticated machine learning techniques in model construction can help with some of these issues. However, the current dearth of appropriate demonstration tools is a disadvantage of the machine learning models. Consequently, researchers need to select the right model creation techniques based on the particular circumstance. Although all of the models performed well overall, there was still a significant chance of bias. The number of events, processing of continuous variables, methods for selecting predictor variables, data complexity, model calibration and fitting, time interval between assessment of predictor variables and outcome determination, and study design (cohort or case–control study) all require improvement. Future research should concentrate on developing new models through larger, rigorously designed studies including multicenter external validations and improved reporting transparency, in order to increase the prediction models’ usefulness for evaluating discharge disposition risks in stroke patients. With the continuous progress in research methods and data processing technologies, the model will be continuously optimized and adjusted to better adapt to clinical needs, and thus provide a more accurate risk assessment tool in clinical practice.

## 5 Strengths and limitations

This review is the first to scientifically and systematically evaluate the risk prediction models for discharge disposition in stroke patients through PROBAST and TRIPOD guidelines based assessment quality and ROB. We detail the characteristics of existing models and also summarize the most common predictors of, providing a valuable reference for healthcare personnel’ targeted care of high-risk patients. This review may be limited in a number of ways. First, our analysis was limited to papers published in English and Chinese, which would have introduced language bias by excluding pertinent studies published in other languages that might have included useful data. Second, there is currently a dearth of research on stroke discharge disposition prediction models in mainland China, with the majority of studies being carried out in nations like the US, Japan, and Australia. This could restrict the findings’ applicability to other geographical areas and call for modifications when using these models in various contexts. Third, a thorough evaluation of the predictive models was limited by certain research’ reporting of only specific performance indicators. Lastly, our meta-analysis only included five validated models from five investigations because of methodological discrepancies and inadequate data. This restriction might have diminished the efficacy of bias evaluations and prevented additional investigation into the causes of study heterogeneity. These problems, however, partly reflect the methodological and reporting difficulties in developing and validating these models rather than affecting the evaluation of the models themselves. Future research requires more rigorous methodology and transparent reporting.

## 6 Conclusion

This systematic review included 14 studies and 22 models, systematically summarizing the features of these models. The findings indicated that the overall effect of models was good, but there was still a high risk of bias, and most lacked external validation. Therefore, the clinical application effect of the models needs further validation. Future studies priority should be given to assessing the applicability of the models, adhering to strict methodological standards, familiar the PROBAST checklist and adhering to the reporting guidelines outlined in the TRIPOD statement to improve the quality of future studies. In addition, we can develop, apply and optimize the prediction model of discharge disposition based on clinical practice by using data mining and AI technology, which could help to enhance the discharge procedure.

## Data Availability

The original contributions presented in the study are included in the article/Supplementary material, further inquiries can be directed to the corresponding authors.

## Footnotes

^1^ https://www.zotero.org/
