# Supplementary material for: Risk prediction models for discharge disposition in patients with stroke: a systematic review and meta-analysis
Source: Front Neurol. 2025 Oct 7;16:1637606. doi: 10.3389/fneur.2025.1637606 (PMC12537401; doi:10.3389/fneur.2025.1637606)
Supplement: Supplementary file 1 [file Table_1.docx]

Supplementary Material

# Supplementary Data

**Table S1.**Electronic search strategy

Search date: September 30, 2024

**| PubMed：187**

#1 "Stroke"[Mesh]

#2 stroke* OR apoplexy* OR cerebrovascular accident OR brain vascular accident[Title/Abstract]

#3 #1 OR #2

#4 "Patient Discharge"[Mesh]

#5 patient discharge OR Post-hospital[Title/Abstract]

#6 #4 OR #5

#7 Disposition* OR outcome* OR destination* OR placement* OR location* OR site* OR agency* OR referral* OR continuation*[Title/Abstract]

#8 "Risk factors"[Mesh]

#9 Risk factors OR risk score OR prediction model OR predictors OR

influence factors[Title/Abstract]

#10 #8 OR #9

#11 #3 AND #6 AND#7 AND #10

**| Web of Science：196**

#1 (TS=(Stroke)) OR AB=(stroke*) OR AB=(apoplexy*) OR AB=(cerebrovascular accident) OR AB=(brain vascular accident)

#2 (TS=(patient discharge )) OR AB=(Post-hospital)

#3 (TS=(Disposition)) OR AB=(Disposition*) OR AB=(outcome*) OR AB=(destination*) OR AB=( placement*) OR AB=(location*) OR AB=(site*) OR AB=( agency* ) OR AB=( referral* ) OR AB=(continuation*)

#4 (TS=(Risk factors)) OR AB=(Risk factors) OR AB=(risk score) OR AB=

(prediction model) OR AB=(predictors) OR AB=(influence factors)

#5 #1 AND #2 AND #3 AND #4

**| Embase：2366**

#1 'Stroke'/exp

#2 'Stroke' OR 'apoplexy*' OR 'cerebrovascular accident' OR 'brain vascular accident':ab,ti

#3 #1 OR #2

#4 'patient discharge'/exp

#5 'patient discharge' OR 'Post-hospital':ab,ti

#6 #4 OR #5

#7 'Disposition'/exp

#8 'Disposition' OR 'outcome*' OR 'destination*' OR 'placement*' OR 'location*' OR 'site*' OR 'agency*' OR 'referral*' OR 'continuation*':ab,ti

#9 #7 OR #8

#9 'Risk factors'/exp

#10 'Risk factors' OR 'risk score' OR 'Prediction model' OR 'predictors' OR 'influence factors':ab,ti

#11 #9 OR #10

#12 #3 AND #6 AND #9AND #11

**| CINAHL:137**

#1 SU Stroke OR AB Stroke OR AB apoplexy* OR AB cerebrovascular accident OR AB brain vascular accident

#2 SU patient discharge OR AB patient discharge OR AB Post-hospital

#3 SU Disposition OR AB Disposition OR AB outcome* OR AB destination* OR AB placement* OR AB location* OR AB referral* OR AB continuation*

#4 SU Risk factors OR AB Risk factors OR AB risk score OR AB Prediction model OR AB predictors OR AB influence factors

#5 #1 AND #2 AND #3AND #4

**|The Cochrane library: 400**

#1 MeSH: Stroke

#2 (Stroke) OR (apoplexy*) OR (cerebrovascular accident ) OR (brain vascular accident):ti,ab,kw

#3 #1 OR #2

#4 MeSH: patient discharge

#5 (patient discharge) OR (Post-hospital ):ti,ab,kw

#6 #4 OR #5

#7 MeSH: Disposition

#8 (Disposition) OR (outcome*) OR (destination*) OR (placement*) OR (location*) OR (referral*) OR (continuation*):ti,ab,kw

#9 #7 OR #8

#10 MeSH: Risk factors

#11 (Risk factors) OR (risk score) OR (Prediction model) OR (predictors) OR (influence factors):ti,ab,kw

#12 #10 OR #11

#13 #3 AND #6 AND #9 AND #12

# Supplementary Figures and Tables

## Supplementary Figures


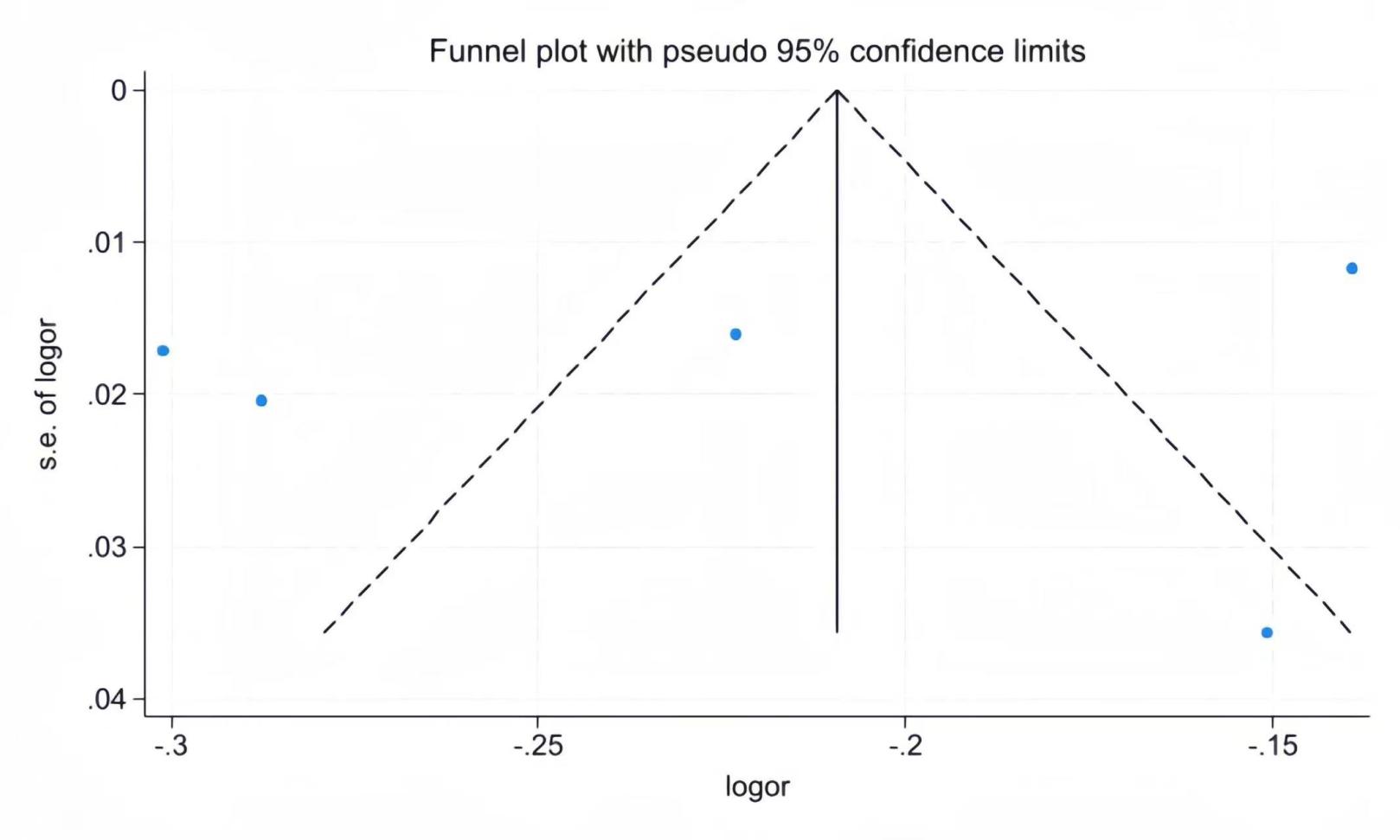


Figure 1. Funnel plot.

## Supplementary Tables

**Table S2.** PROBAST results of the included studies.

| **Included literature(year)** | **Bias risk assessment** | | | | **Applicability assessment** | | | **Overall assessment** | |
| --- | --- | --- | --- | --- | --- | --- | --- | --- | --- |
|  | **Study**  **subjects** | **Predictors** | **Results** | **Analysis** | **Study**  **subjects** | **Predictors** | **Results** | **Bias risk** | **Applicability** |
| Lensky et al.（2024） | + | + | + | － | + | + | + | － | + |
| Cui et al.（2024） | + | + | + | － | + | + | + | － | + |
| Veerbeek et al.（2022） | + | － | + | － | + | + | + | － | + |
| Ito et al.（2022） | + | － | － | － | + | + | + | － | + |
| Itaya et al.（2022） | + | + | + | － | + | + | + | － | + |
| Cho et al.（2021） | － | + | － | － | + | + | + | － | + |
| Berker et al.（2020） | + | + | + | － | + | + | + | － | + |
| Kubo et al.（2020） | + | + | + | － | + | + | + | － | + |
| Kim et al.（2020） | － | + | － | － | － | + | + | － | － |
| Itaya et al.（2017） | + | + | + | － | + | + | + | － | + |
| Ouellette et al.(2015） | + | + | + | － | + | + | + | － | + |
| Béjot et al.(2015) | + | + | + | － | + | + | + | － | + |
| Stineman et al.（2014） | + | + | + | － | － | + | + | － | － |
| Brauer et al.（2008） | + | + | － | － | + | + | + | － | + |

PROBAST, Prediction model Risk Of Bias Assessment Tool; "+" indicates low risk of bias/high applicability; "-" indicates high risk of bias/low applicability; "?" indicates unclear.

**Table S3.**Risk of Bias Assessment for the Included Studies.

| **Auther(year)** | **Participants** | **Predictors** | **Outcomes** | **Analysis** | **Overall ROB** |
| --- | --- | --- | --- | --- | --- |
| Lensky et al.（2024） | Low | Low | Low | High | High |
| Cui et al.（2024） | Low | Low | Low | High | High |
| Veerbeek et al.（2022） | Low | High | Low | High | High |
| Ito et al.（2022） | Low | High | High | High | High |
| Itaya et al.（2022） | Low | Low | Low | Unclear | High |
| Cho et al.（2021） | High | Low | High | High | High |
| Berker et al.（2020） | Low | Low | Low | High | High |
| Kubo et al.（2020） | Low | Low | Low | Unclear | High |
| Kim et al.（2020） | High | Low | High | High | High |
| Itaya et al.（2017） | Low | Low | Low | High | High |
| Ouellette et al.(2015） | Low | Low | Low | Unclear | High |
| Béjot et al.(2015) | Low | Low | Low | High | High |
| Stineman et al.（2014） | Low | Low | Low | Unclear | High |
| Brauer et al.（2008） | Low | Low | High | High | High |

**Table S4.** TRIPOD checklist.

|  | **1. Title(D;V)** | **2. Abstract: summary(D;V)** | **3a. Introduction: the medical context(D;V)** | **3b. Objectives(D;V)** | **4a. Methods: study design(D;V)** | **4b. Key study dates(D;V)** | **5a. Study setting(D;V)** | **5b. Eligibility criteria(D;V)** | **5c. Treatment, if relevant(D;V)** | **6a. Outcome definition(D;V)** | **6b. Blind assessment of outcome(D;V)** | **7a. Definition of predictors(D;V)** | **7b. Blind assessment of predictors(D;V)** | **8. Study size(D;V)** | **9. Handling missing data(D;V)** | **10a.Handling predictors(D)** | **10b. Model development procedure(D)** | **10c.Describe how the predictions are calculated(V)** | **10d. Assessing model performance(D;V)** | **10e.Validate model updates(V)** | **11. Risk groups(D;V)** | **12.Validation differs from the modeling data(V)** | \| **13a. Results: flow of participant selection(D;V)** \| \| --- \| | **13b. Participant characteristics(D;V)** | **13c.variables were compared with the data (V)** | **14a. Number of outcome(D)** | **14b. Unadjusted association, if done(D)** | **15a. Full model presentation(D)** | **15b. How to use the model(D)** | **16. Performance measures(D;V)** | **17.Report the model update results(V)** | **18. Discussion: study limitations(D;V)** | **19a.Discuss reference data(V)** | **19b. Overall interpretation(D;V)** | **20. Potential clinical use(D;V)** | **21. Supplementary information(D;V)** | **22. Funding(D;V)** | |
| --- | --- | --- | --- | --- | --- | --- | --- | --- | --- | --- | --- | --- | --- | --- | --- | --- | --- | --- | --- | --- | --- | --- | --- | --- | --- | --- | --- | --- | --- | --- | --- | --- | --- | --- | --- | --- | --- | --- | --- |
| Lensky et al.（2024） | 2 | 1 | 1 | 1 | 1 | 1 | 1 | 1 | 1 | 1 | 3 | 1 | 1 | 1 | 1 | 1 | 1 | 3 | 1 | 3 | 2 | 1 | 1 | 1 | 1 | 1 | 3 | 1 | 2 | 1 | 1 | 1 | 1 | 1 | 1 | 1 | 1 | |
| Cui et al.（2024） | 2 | 1 | 1 | 1 | 1 | 1 | 1 | 1 | 1 | 1 | 3 | 1 | 1 | 1 | 1 | 1 | 1 | 3 | 1 | 3 | 2 | 1 | 1 | 1 | 1 | 1 | 1 | 1 | 1 | 1 | 1 | 1 | 1 | 1 | 1 | 1 | 1 | |
| Veerbeek et al.（2022） | 1 | 1 | 1 | 1 | 1 | 1 | 1 | 1 | 1 | 1 | 3 | 1 | 1 | 1 | 1 | 1 | 1 | 2 | 1 | 2 | 2 | 1 | 1 | 1 | 1 | 1 | 3 | 1 | 1 | 1 | 1 | 1 | 1 | 1 | 1 | 1 | 1 | |
| Ito et al.（2022） | 1 | 1 | 1 | 1 | 1 | 1 | 1 | 1 | 1 | 1 | 3 | 1 | 1 | 1 | 1 | 1 | 2 | 3 | 1 | 3 | 2 | 1 | 1 | 1 | 1 | 1 | 2 | 1 | 1 | 1 | 1 | 1 | 1 | 1 | 1 | 1 | 1 | |
| Itaya et al.（2022） | 1 | 1 | 1 | 1 | 1 | 1 | 1 | 1 | 1 | 1 | 3 | 1 | 1 | 1 | 2 | 1 | 3 | 1 | 1 | 2 | 2 | 1 | 2 | 1 | 1 | 1 | 1 | 1 | 2 | 1 | 1 | 1 | 1 | 1 | 1 | 1 | 1 | |
| Cho et al.（2021） | 1 | 1 | 1 | 1 | 1 | 1 | 1 | 1 | 1 | 1 | 3 | 1 | 1 | 2 | 1 | 1 | 1 | 3 | 1 | 3 | 1 | 1 | 1 | 2 | 1 | 1 | 1 | 3 | 2 | 2 | 2 | 1 | 3 | 1 | 1 | 1 | 1 | |
| Berker et al.（2020) | 2 | 1 | 1 | 1 | 1 | 1 | 1 | 1 | 1 | 1 | 3 | 1 | 1 | 1 | 1 | 1 | 1 | 3 | 1 | 3 | 2 | 1 | 1 | 1 | 1 | 1 | 1 | 3 | 2 | 1 | 1 | 1 | 1 | 1 | 1 | 1 | 1 | |
| Kubo et al.（2020） | 1 | 1 | 1 | 1 | 1 | 1 | 1 | 1 | 1 | 1 | 3 | 1 | 1 | 1 | 1 | 1 | 1 | 3 | 1 | 3 | 1 | 1 | 1 | 1 | 1 | 1 | 1 | 3 | 2 | 1 | 1 | 1 | 1 | 1 | 1 | 1 | 1 | |
| Kim et al.（2020） | 1 | 1 | 1 | 1 | 1 | 1 | 1 | 1 | 1 | 1 | 3 | 1 | 1 | 1 | 1 | 1 | 2 | 2 | 1 | 2 | 1 | 1 | 1 | 1 | 1 | 1 | 1 | 1 | 2 | 1 | 1 | 1 | 1 | 1 | 1 | 1 | 1 | |
| Itaya et al.（2017） | 1 | 1 | 1 | 1 | 1 | 1 | 1 | 1 | 1 | 1 | 3 | 1 | 1 | 1 | 1 | 1 | 1 | 3 | 1 | 3 | 1 | 1 | 1 | 2 | 1 | 1 | 1 | 1 | 2 | 1 | 1 | 1 | 1 | 1 | 1 | 1 | 1 | |
| Ouellette et al.（2015） | 2 | 1 | 1 | 1 | 1 | 1 | 1 | 1 | 1 | 1 | 3 | 1 | 1 | 1 | 1 | 1 | 2 | 3 | 1 | 3 | 1 | 1 | 1 | 1 | 1 | 1 | 1 | 1 | 2 | 1 | 1 | 1 | 1 | 1 | 1 | 1 | 1 | |
| Béjot et al.（2015） | 2 | 1 | 1 | 1 | 1 | 1 | 1 | 1 | 1 | 1 | 3 | 1 | 1 | 1 | 1 | 1 | 2 | 2 | 1 | 2 | 1 | 1 | 1 | 1 | 1 | 1 | 1 | 1 | 2 | 1 | 1 | 1 | 1 | 1 | 1 | 1 | 1 | |
| Stineman et al.（2014） | 1 | 1 | 1 | 1 | 1 | 1 | 1 | 1 | 1 | 1 | 3 | 1 | 1 | 1 | 2 | 1 | 1 | 3 | 1 | 3 | 1 | 1 | 1 | 1 | 1 | 1 | 1 | 1 | 1 | 1 | 1 | 1 | 1 | 1 | 1 | 1 | 1 | |
| Brauer et al.（2008） | 2 | 1 | 1 | 1 | 1 | 1 | 1 | 1 | 1 | 1 | 3 | 1 | 1 | 1 | 1 | 1 | 1 | 3 | 1 | 3 | 2 | 1 | 1 | 1 | 1 | 1 | 1 | 1 | 1 | 1 | 1 | 1 | 1 | 1 | 1 | 1 | 1 | |
| 1 indicates Reported, 2 indicates Not reported, 3 indicates Not applicable;D, items relevant to the development of a prediction model; V, items relevant to the validation of a prediction model; D;V, items relevant to both of the development and validation of a prediction model. | | | | | | | | | | | | | | | | | | | | | | | | | | | | | | | | | | | | | |  |

**Table S5.**PRISMA (Preferred Reporting Items for Systematic Reviews and Meta-Analyses) checklist

| **Section and Topic** | **Item #** | **Checklist item** | **Location where item is reported** |
| --- | --- | --- | --- |
| **TITLE** | | |  |
| Title | 1 | Identify the report as a systematic review. | **Manuscript,** Page 1 |
| **ABSTRACT** | | |  |
| Abstract | 2 | See the PRISMA 2020 for Abstracts checklist. | **Manuscript**, Page 1-2 |
| **INTRODUCTION** | | |  |
| Rationale | 3 | Describe the rationale for the review in the context of existing knowledge. | **Manuscript**, Introduction |
| Objectives | 4 | Provide an explicit statement of the objective(s) or question(s) the review addresses. | **Manuscript**, Introduction |
| **METHODS** | | |  |
| Eligibility criteria | 5 | Specify the inclusion and exclusion criteria for the review and how studies were grouped for the syntheses. | **Manuscript**, 2.2 Inclusion and exclusion criteria |
| Information sources | 6 | Specify all databases, registers, websites, organisations, reference lists and other sources searched or consulted to identify studies. Specify the date when each source was last searched or consulted. | **Manuscript**, 2.1 Search strategy |
| Search strategy | 7 | Present the full search strategies for all databases, registers, and websites, including any filters and limits used. | **Manuscript,** 2.1  Search strategy  **Supplementary**  Table S1.Electronic search strategy |
| Selection process | 8 | Specify the methods used to decide whether a study met the inclusion criteria of the review, including how many reviewers screened each record and each report retrieved, whether they worked independently, and if applicable, details of automation tools used in the process. | **Manuscript**, 2.3 Study selection |
| Data collection process | 9 | Specify the methods used to collect data from reports, including how many reviewers collected data from each report, whether they worked independently, any processes for obtaining or confirming data from study investigators, and if applicable, details of automation tools used in the process. | **Manuscript** 2.3. Study selection and screening |
| Data items | 10a | List and define all outcomes for which data were sought. Specify whether all results that were compatible with each outcome domain in each study were sought (e.g., for all measures, time points, analyses), and if not, the methods used to decide which results to collect. | **Manuscript**, 2.4. Data extraction |
|  | 10b | List and define all other variables for which data were sought (e.g., participant and intervention characteristics, funding sources). Describe any assumptions made about any missing or unclear information. | **Manuscript**, 2.4. Data extraction |
| Study risk of bias assessment | 11 | Specify the methods used to assess risk of bias in the included studies, including details of the tool(s) used, how many reviewers assessed each study and whether they worked independently, and if applicable, details of automation tools used in the process. | **Manuscript**, 2.5.Risk of bias and applicability assessment |
| Effect measures | 12 | Specify for each outcome the effect measure(s) (e.g., risk ratio, mean difference) used in the synthesis or presentation of results. | **Manuscript**, 2.6. Data synthesis and statistical analysis |
| Synthesis methods | 13a | Describe the processes used to decide which studies were eligible for each synthesis (e.g., tabulating the study intervention characteristics and comparing against the planned groups for each synthesis (item #5)). | **Manuscript**, 2.6. Data synthesis and statistical analysis |
|  | 13b | Describe any methods required to prepare the data for presentation or synthesis, such as handling of missing summary statistics, or data conversions. | **Manuscript**, 2.6. Data synthesis and statistical analysis |
|  | 13c | Describe any methods used to tabulate or visually display results of individual studies and syntheses. | **Manuscript**, 2.6. Data synthesis and statistical analysis |
|  | 13d | Describe any methods used to synthesise results and provide a rationale for the choice(s). If meta-analysis was performed, describe the model(s), method(s) to identify the presence and extent of statistical heterogeneity, and software package(s) used. | **Manuscript**, 2.6. Data synthesis and statistical analysis |
|  | 13e | Describe any methods used to explore possible causes of heterogeneity among study results (e.g., subgroup analysis, meta-regression). | Not applicable |
|  | 13f | Describe any sensitivity analyses conducted to assess robustness of the synthesised results. | **Manuscript**, 2.5.Risk of bias and applicability assessment |
| Reporting bias assessment | 14 | Describe any methods used to assess risk of bias due to missing results in a synthesis (arising from reporting biases). | Not applicable |
| Certainty assessment | 15 | Describe any methods used to assess certainty (or confidence) in the body of evidence for an outcome. | Not applicable |
| **RESULTS** | | |  |
| Study selection | 16a | Describe the results of the search and selection process, from the number of records identified in the search to the number of studies included in the review, ideally using a flow diagram. | **Manuscript**, 3. Results; Figure 1 |
|  | 16b | Cite studies that might appear to meet the inclusion criteria, but which were excluded, and explain why they were excluded. | Not applicable |
| Study characteristics | 17 | Cite each included study and present its characteristics. | **Manuscript**, 3.1. Study selection, Table 2&Table 3 |
| Risk of bias in studies | 18 | Present assessments of risk of bias for each included study. | **Manuscript**, 3.4. Results of quality assessment，Figure 2，**Supplementary** Table S2&3 |
| Results of individual studies | 19 | For all outcomes, present, for each study: (a) summary statistics for each group (where appropriate) and (b) an effect estimate and its precision (e.g. confidence/credible interval), ideally using structured tables or plots. | **Manuscript**, 3.2. Study characteristics, Table 2&Table 3 |
| Results of syntheses | 20a | For each synthesis, briefly summarise the characteristics and risk of bias among contributing studies. | **Manuscript**, 3.4. Results of quality assessment，Figure 2，**Supplementary** Table S2&3 |
|  | 20b | Present results of all statistical syntheses conducted. If meta-analysis was done, present for each the summary estimate and its precision (e.g., confidence/credible interval) and measures of statistical heterogeneity. If comparing groups, describe the direction of the effect. | Not applicable |
|  | 20c | Present results of all investigations of possible causes of heterogeneity among study results. | Not applicable |
|  | 20d | Present results of all sensitivity analyses conducted to assess the robustness of the synthesised results. | Not applicable |
| Reporting biases | 21 | Present assessments of risk of bias due to missing results (arising from reporting biases) for each synthesis assessed. | Not applicable |
| Certainty of evidence | 22 | Present assessments of certainty (or confidence) in the body of evidence for each outcome assessed. | Not applicable |
| **DISCUSSION** | | |  |
| Discussion | 23a | Provide a general interpretation of the results in the context of other evidence. | **Manuscript**, 4. Discussion |
|  | 23b | Discuss any limitations of the evidence included in the review. | **Manuscript**, .5. Strengths and Limitations |
|  | 23c | Discuss any limitations of the review processes used. | **Manuscript**, 5. Strengths and Limitations |
|  | 23d | Discuss implications of the results for practice, policy, and future research. | **Manuscript**, 4.3. Implications for Clinical Practice |
| **OTHER INFORMATION** | | |  |
| Registration and protocol | 24a | Provide registration information for the review, including register name and registration number, or state that the review was not registered. | **Manuscript**, 2. Methods |
|  | 24b | Indicate where the review protocol can be accessed, or state that a protocol was not prepared. | **Manuscript**, 2. Methods |
|  | 24c | Describe and explain any amendments to information provided at registration or in the protocol. | Not applicable |
| Support | 25 | Describe sources of financial or non-financial support for the review, and the role of the funders or sponsors in the review. | Title page |
| Competing interests | 26 | Declare any competing interests of review authors. | Title page |
| Availability of data, code, and other materials | 27 | Report which of the following are publicly available and where they can be found; template data collection forms; data extracted from included studies; data used for all analyses; analytic code; any other materials used in the review. | Not applicable |

**Table S6.** CHARMS checklist.

| **Domain** | **Key items** |
| --- | --- |
|  |  |
| **SOURCE OF DATA** | Source of data (e.g., cohort, case-control, randomized trial participants, or registry data) |
| **PARTICIPANTS** | Participant eligibility and recruitment method (e.g., consecutive participants, location, number of centers, setting, inclusion and exclusion criteria) |
|  | Participant description |
|  | Details of treatments received, if relevant |
|  | Study dates |
| **OUTCOME(S) TO BE PREDICTED** | Definition and method for measurement of outcome |
|  | Was the same outcome definition (and method for measurement) used in all patients? |
|  | Type of outcome (e.g., single or combined endpoints) |
|  | Was the outcome assessed without knowledge of the candidate predictors (i.e., blinded)? |
|  | Were candidate predictors part of the outcome (e.g., in panel or consensus diagnosis)? |
|  | Time of outcome occurrence or summary of duration of follow-up |
| **CANDIDATE PREDICTORS (OR INDEX TESTS)** | Number and type of predictors (e.g., demographics, patient history, physical examination, additional testing, disease characteristics) |
|  | Definition and method for measurement of candidate predictors |
|  | Timing of predictor measurement (e.g., at patient presentation, at diagnosis, at treatment initiation) |
|  | Were predictors assessed blinded for outcome, and for each other (if relevant)? |
|  | Handling of predictors in the modelling (e.g., continuous, linear, non-linear transformations or categorised) |
| **SAMPLE SIZE** | Number of participants and number of outcomes/events |
|  | Number of outcomes/events in relation to the number of candidate predictors (**Events Per Variable**) |
| **MISSING DATA** | Number of participants with any missing value (include predictors and outcomes) |
|  | Number of participants with missing data for each predictor |
|  | Handling of missing data (e.g., complete-case analysis, imputation, or other methods) |
| **MODEL DEVELOPMENT** | Modelling method (e.g., logistic, survival, neural network, or machine learning techniques) |
|  | Modelling assumptions satisfied |
|  | Method for selection of predictors **for inclusion** in multivariable modelling (e.g., all candidate predictors, pre-selection based on unadjusted association with the outcome) |
|  | Method for selection of predictors **during multivariable modelling** (e.g., full model approach, backward or forward selection) and criteria used (e.g., p-value, Akaike Information Criterion) |
|  | Shrinkage of predictor weights or regression coefficients (e.g., no shrinkage, uniform shrinkage, penalized estimation) |
| **MODEL PERFORMANCE** | Calibration (calibration plot, calibration slope, Hosmer-Lemeshow test) and Discrimination (C-statistic, D-statistic, log-rank) measures with confidence intervals |
|  |  |
|  | Classification measures (e.g., sensitivity, specificity, predictive values, net reclassification improvement) and whether a-priori cut points were used |
| **MODEL EVALUATION** | Method used for testing model performance: development dataset only (random split of data, resampling methods e.g. bootstrap or cross-validation, none) or separate external validation (e.g. temporal, geographical, different setting, different investigators) |
|  | In case of poor validation, whether model was adjusted or updated (e.g., intercept recalibrated, predictor effects adjusted, or new predictors added) |
| **RESULTS** | Final and other multivariable models (e.g., basic, extended, simplified) presented, including predictor weights or regression coefficients, intercept, baseline survival, model performance measures (with standard errors or confidence intervals) |
|  | Any alternative presentation of the final prediction models, e.g., sum score, nomogram, score chart, predictions for specific risk subgroups with performance |
|  | Comparison of the distribution of predictors (including missing data) for development and validation datasets |
| **INTERPRETATION AND DISCUSSION** | Interpretation of presented models (confirmatory, i.e., model useful for practice versus exploratory, i.e., more research needed) |
|  | Comparison with other studies, discussion of generalizability, strengths and limitations. |
